# A Discovery of the True Cause of the Disease Called by the People Trembles or Milk-sickness

**Published:** 1843-01

**Authors:** 


### A DISCOVERY OF THE TRUE CAUSE OF THE DISEASE CALLED BY THE PEOPLE TREMBLES OR MILK-SICKNESS.

This is the title of a pamphlet of forty-eight pages, from the Louisville press, laid on our table by its author, Mr. Ernst Heeringen. Our city is prolific in this milky literature—ourselves having led off in the spring of 1840; followed by Dr. Seaton, in a few months, with a production of nearly the same form and size; to which is now added a third, by Mr. Heeringen, bearing so much *prima facie* resemblance to our own, as to suggest the pleasing anticipation that we have founded a new school of medical literature. Our *followers* (if such they are) have, however, already got *before* us; as each has *discovered* the remote cause of this mysterious disorder—under which man turns *sick* and his domestic animals *tremble*. Unfortunately, however, our followers differ a *little*, as to the precise agent, one having ascertained that it is arsenic, the other malaria. So there they stand—"*Each claiming truth and truth*"—but we shall not finish the quotation, as we wish to treat all scientific inquirers with respect.

From the very dawn of the bright day of *conjecture*, which has so long beamed upon this subject, arsenic and malaria have been accused of what our rival authors have lately charged upon them; but although so often indicted, the profession have not yet declared them guilty. *Should* either of them be convicted, the present complainants would have no very strong claims to the merit of discovery; except in the *evidence* by which the conviction was effected. Of Doctor Seaton's success, in presenting new testimony, we spoke at the time his pamphlet made its appearance; and are not aware, that he has since laid any thing conclusive before the profession. The style in which Dr. Seaton and his pamphlet are spoken of by Mr. H., shows that *his* "milk of human kindness" has got in it a small "sprinkle" of acrid poison. We extract a couple of paragraphs, as "a caution" to future speculatists.

"Dr. Seaton is also wrong, when he says, that cows that are well salted do not go so much to the springs, and therefore receive less arsenic. Now every farrier and almost every farmer knows, that salt purges gently, and dissolves, and thus proves a deductor of impurities contained in the prima via; but also they do know, that salting makes the animals drink more than they otherwise would have done. But I say, let the shoemaker stick to his last. For the correctness of this, I refer the reader to any good work on veterinary practice."

"Arsenic is one of the most powerful mineral poisons. Who will be surprised, therefore, when Dr. Seaton says, that, in giving to three cows sixty grains of this mineral in one day, and twenty-four grains at a dose, two of them died in forty-eight hours after receiving the first dose, and one of them was very near dead. But he has proved nothing else by this experiment, except, that arsenic is apt to kill cows—a thing which every schoolboy knew before; inasmuch as the calves that were sucking the cows during the time of these experiments never felt the worse for it, and the hogs and dogs that ate the flesh of the dead cows were nothing the less incommoded after their hearty meal. I would therefore take this opportunity to say, that it would probably have been much better if Dr. Seaton had made the above experiments previously, instead of making them after having frightened the community by his arsenic pamphlet."

If Dr. Seaton felt himself so much injured, by *our* former decorous citation of a few facts, which stood in the way of his adopted hypothesis, we fear he will be quite "out of patience" with Mr. Heeringen, to whose pamphlet we must devote the remainder of our article.

Mr. H. is a German veterinary practitioner, whose style is sufficiently accurate for the object he has in view, and whose inquiries into the diseases of our domestic animals, give him some fitness for the special investigation he has undertaken. We cannot, however, say quite so much for his logic. Having shown, as he conceives, that Trembles neither arise from a mineral, vegetable, nor animal poison, he comes boldly up to the conclusion, that it *must* be produced by malaria. The argument is, that it exists, it has a cause, nothing else but malaria can produce it, and, therefore, malaria does produce it, *quod erat,* &c. It was in tracing its symptomic analogies, however, that he got the first insight into its ætiology. These, as he informs us, place it under the head of Anthrax fevers (*febres ataxo adynamica*) of Dietrich. As a nosological appellation he proposes the following as "most proper."

"Epizootica Americana aut Febris ataxo acutissimus cum caractore contagiose—or in English, Trembles—which is as good a denomination as any that could possibly be made use of. Milk-sickness, or sick stomach, should be baptized Epidemica Americana Secundaria."

For the analogies by which our author, has, to borrow his own language, "established on philosophical principles the diagnosis of Trembles," we must refer to the pamphlet itself; proceeding to state, what he has added to his a priori reasonings, from the *a posteriori*. This can be soon done, for we have not been able to find a single fact, or one, at least, not repeatedly urged by previous advocates of the miasmatic hypothesis. On this point, we were unpleasantly disappointed, for as he is evidently an observing man, and has spent much time among the farmers, on either bank of the Ohio, we had hoped, that he would have contributed some new observations of a valuable kind. It is but just, however, that he should be allowed to speak for himself.

"By analogy we have become convinced that Trembles are an epizootic malady; and by comparing their symptoms with those of joint murrain, according to Dr. Dietrich's excellent work, we have proved that Trembles belong to the Anthrax family; we have further given ample proof, that Trembles are caused, neither by a vegetable nor a mineral poison, but on the contrary by epizootic or miasmatic impurities, and therefore leave this portion of our task, with the assurance that it will not be long, before all, that are interested in this matter, cheerfully agree, that our opinion is based upon truth and fact."

This brings us to our authors means of prevention, which we are happy to say, even when employed but in part, have for many years, in all portions of the west, been found amply sufficient to prevent the disease. As he is well acquainted with veterinary hygieen, we commend to our agriculturalists all that he has said; and will vouch, that if they carry out his directions, their cattle will not only remain free from Trembles, but some other maladies, which are the offspring of a negligent rural economy.

"Dr. Drake says, in vol. iv., No. v, p. 370 of the Western Medical Journal, that, when the cause of Trembles is discovered, the axe and plough will prove to be the only means by which the progress of the disease can be stopped.

"This assertion is based upon experience; but we know, from the statement of Mr. Walker Hawes and others, that the axe alone is sufficient to arrest the Trembles in a short time. However, farmers would do well to proceed as follows:

"*First.* All the trees that can be dispensed with, ought to be cut down and burned; so as to prevent their decaying in the place, and thus produce noxious malaria.

"*Second.* Others that are wanted ought to be deadened.

"*Third.* The buckeye, burr-oak, black-walnut, and maples ought to be cut down, principally, because their leaves do more damage than those of others.

"*Fourth.* In low and marshy places, all the trees without distinction ought to be cut down and burnt or deadened.

"*Fifth.* In dry weather, fire ought to be set to the leaves and wood lying about in the forest.

"*Sixth.* Dead animals ought to be buried immediately, and not suffered to impregnate the air with putrefaction, or to be eaten by hogs and dogs.

"*Seventh.* Farmers ought to salt their stock every day regularly; for the salt cools, dissolves, and opens their bowels, and thus frequently removes the causes of some diseases.

"*Eighth.* In districts where Trembles prevail, and during the sickly season, the milk-cow ought to be kept in a fenced lot, in which the timber has partly been deadened or cut down.

"*Ninth.* The springs ought to be kept clean, and an outlet for the water ought to be made: this will prevent the water from overflowing the ground in the vicinity of the spring.

"*Tenth.* Ponds must be fenced in, or the water drained off, if possible.

"*Eleventh.* Animals that are kept up where they cannot procure themselves water, must be watered regularly; and for this purpose a trough is best, set in the place where they are, and filled with good water.

"*Twelfth.* During the hot season, animals ought to be bathed; and where this cannot be done, they ought, from time to time, to be sprinkled with fresh water.

"*Thirteenth.* Every animal ought, once in a month, to be purged with glauber salts.

"*Fourteenth.* Animals must not be suffered to go to places that have recently been overflowed, or where there is a good deal of stagnant water.

"*Fifteenth.* When there are meal or honey dews falling, animals must be kept under fence.

"*Sixteenth.* Animals ought not to be permitted to lie out during the night, but kept in stables or under fence, from sundown until next morning.

"*Seventeenth.* It is of great advantage, if animals, in the hot season, are kept in shady places, and not exposed to the rays of the sun.

"*Eighteenth.* Cows ought to be bled at least once every summer."

Our author has devoted two pages, at the close of his book, to the treatment of Trembles, which, he tells us, contain the results of his experience. They may be stated, to consist in bleeding, purging, rowelling, and the cold effusion, for the details of which we refer to the pamphlet itself.

We understand, that Mr. H. is a candidate for the premium offered by our Legislature, for the discovery of the remote cause of Trembles and Milk-sickness; and we sincerely hope he will continue his researches, until the results shall place it in his hands. D.

